# MATria: a unified centrality algorithm

**DOI:** 10.1186/s12859-019-2820-7

**Published:** 2019-06-06

**Authors:** Trevor Cickovski, Vanessa Aguiar-Pulido, Giri Narasimhan

**Affiliations:** 1 Bioinformatics Research Group (BioRG) & Biomolecular Sciences Institute, School of Computing & Information Sciences, Florida International University, 11200 SW 8th St, Miami, 33199 FL USA; 2 Center for Neurogenetics, Weill Cornell Medical College, New York, 10021 NY USA

**Keywords:** Centrality, Networks, Iteration, Graphics Processing Unit (GPU)

## Abstract

**Background:**

Computing *centrality* is a foundational concept in social networking that involves finding the most “central” or important nodes. In some biological networks defining *importance* is difficult, which then creates challenges in finding an appropriate centrality algorithm.

**Results:**

We instead generalize the results of any *k* centrality algorithms through our iterative algorithm MATRIA, producing a single ranked and *unified* set of central nodes. Through tests on three biological networks, we demonstrate evident and balanced correlations with the results of these *k* algorithms. We also improve its speed through GPU parallelism.

**Conclusions:**

Our results show iteration to be a powerful technique that can eliminate spatial bias among central nodes, increasing the level of agreement between algorithms with various importance definitions. GPU parallelism improves speed and makes iteration a tractable problem for larger networks.

## Background

The concept of *centrality* is fundamental to social network theory and involves finding the most important or *central* nodes in a social network. There are three core types of *path-based* centrality, each with different definitions of *importance*. *Betweenness* centrality [1] bases importance on the number of shortest paths over all pairs of nodes that run through a node (finding hubs in a network), *closeness* [2] on the overall length of the shortest paths towards all other nodes that start from a node (finding nodes in the “center” of a network), and *degree* [3] on the number of connections. There are also *eigenvector-based* approaches, which solve a system of *n* equations with *n* unknown centrality values for a graph of *n* nodes, applying an eigensolver that eventually converges to the centrality values. *PN-*centrality [4] takes into account a node’s local degree and that of its “friends” and “enemies”. Google’s PageRank [5] models centrality by a random walker which probabilistically either moves to a neighbor or someplace random, with centrality values reflecting how often this walker lands upon a node. PageTrust [6] extends PageRank to handle signed networks by incorporating *distrust* between nodes.

Many real-world networks (i.e., airports, search engines) have a clear definition of “importance”, enabling the appropriate centrality algorithm to be chosen. When studying biological networks this can also be true, as has been shown with phylogenetically older metabolites tending to have larger degree in a metabolic network [7], and the removal of highly connected proteins within yeast protein interaction networks tending to be lethal [8]. Other times this is not so certain, as when studying properties such as transitivity in protein interaction networks [9], robustness against mutations in gene networks [10], and finding global regulators in gene regulatory networks [11]. This latter study in particular showed large amounts of disagreement between centrality algorithms in uncovering global regulators in an *E. Coli* gene regulatory network, and along with other studies [12, 13] indicates it is necessary to apply multiple centrality algorithms in situations where “importance” is difficult to define.

**Fig. 1:**
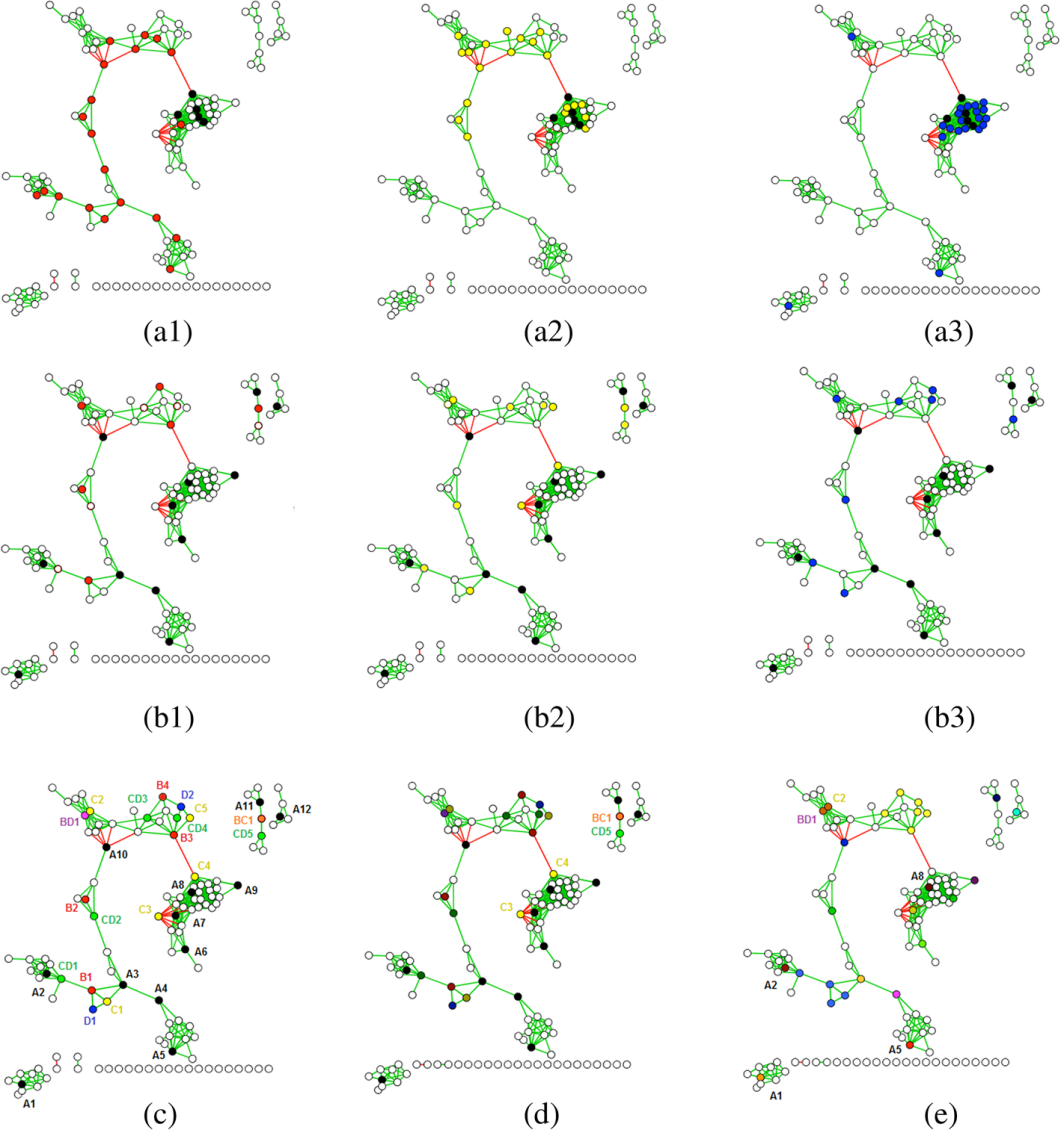
Centrality results on a test microbial co-occurrence network. Top 20% most central nodes found by non-iterative betweenness (**a1**, red), closeness (**a2**, yellow) and degree (**a3**, blue) centrality in a correlation network, with mutual agreements in black. Central nodes found by iterative betweenness (**b1**), closeness (**b2**) and degree (**b3**) centrality on the same network, again with mutual agreements in black. **c** Same network with nodes found by all (black), betweenness only (red), closeness only (yellow), degree only (blue), betweenness and closeness (orange), closeness and degree (green), and betweenness and degree (violet). **d** Final network with all possible disagreements (dark) resolved. **e** Final centrality rankings of nodes and supernodes produced by MATRIA, red nodes are highly ranked, violet low, white zero

**Fig. 2:**
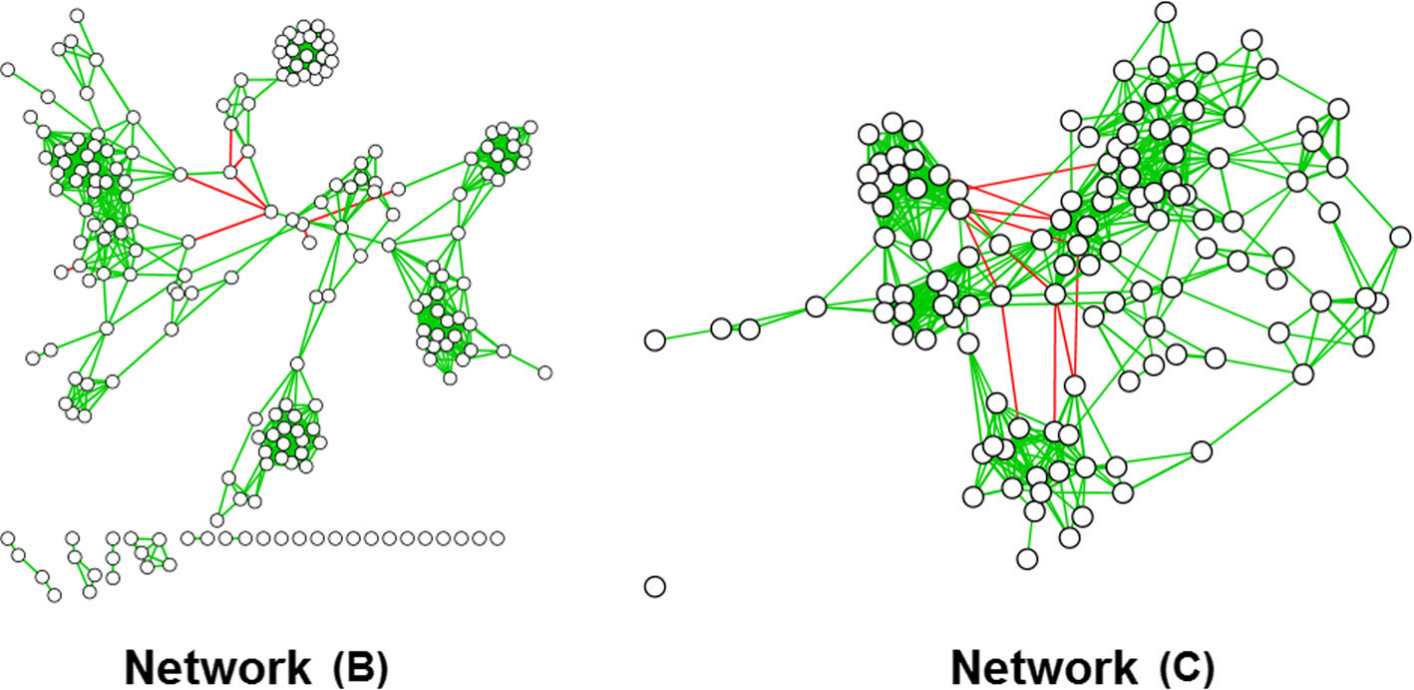
Two other test biological networks. **b** Gene co-expression network from the Pacific Oyster; **c** Less modular microbial co-occurrence network

The challenge in these situations then becomes how to unify results over multiple centrality algorithms that differ in their definitions of “importance” and therefore also their results. Figure 1 shows application of the three path-based approaches to a signed and weighted bacterial co-occurrence network [14], with parts (a1-3) demonstrating minimal similarity between each algorithm’s top 20% most central nodes. To be certain we also tested on the two less modular biological networks shown in Fig. 2, including a Pacific Oyster gene co-expression network (GEO:GSE31012, network B) and a more fully connected bacterial co-occurrence network C. Table 1 shows Spearman correlations between rank vectors from the three path-based approaches (network A is from Fig. 1). Correlation with betweenness and the other two approaches peaked for network B, but went to almost zero for network A (modular) and network C (well-connected). Correlation between degree and closeness was the opposite, peaking for the extremes but low for network B.

**Table 1:** Rank vector correlations between non-iterative centrality algorithms on three signed/weighted biological networks

Algorithm Pairs	A	B	C
Betweenness/Closeness	0.071	0.396	-0.120
Betweenness/Degree	-0.170	0.106	-0.136
Closeness/Degree	0.699	0.256	0.863

Figure 1**a1-3** makes it evident that spatial biases within each algorithm largely contribute to this disagreement. For network A all central nodes were mostly on the same path with betweenness (a1), in the “middle” with closeness (a2), and in the same strongly connected component with degree (a3). The network had 126 nodes, and the three algorithms agreed on only five central nodes (in black) within their top 20%. This naturally leads to the question, if we were to somehow remove spatial bias, would we have more consensus among the results?

We build on a prior algorithm called ATRIA [15], which reduced bias in closeness centrality by applying iteration to identify central nodes spread widely across the network. We used a socio-economic model with node pairs providing a “gain” and a “loss” to each other. We will now apply iteration to other centrality algorithms (which we refer to as *backbones*), and first illustrate stronger agreement between iterative backbones on our biological networks compared to their non-iterative counterparts. We next propose an algorithm MATRIA for unifying disagreements between these iterative backbones, producing a ranked set of central nodes and *supernodes* with multiple central node possibilities. This unified set had good coverage for our networks, with 90-100% of the nodes either in this set or universally agreed as unimportant. We also demonstrate that this rank vector correlates well with those from the iterative backbones, which by consilience [16] supports its reliability. Since iteration is computationally expensive we conclude with a discussion on improving efficiency for large biological networks through the GPU.

### Background: iteration

**Fig. 3:**
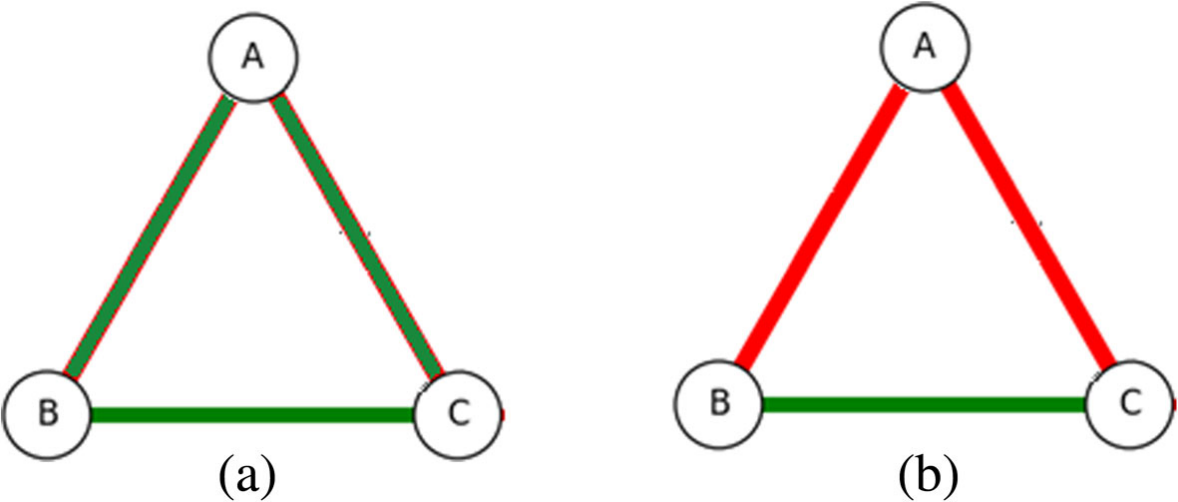
Stable triads, with (**a**) zero and (**b**) two negative edges


With ATRIA we found spatial bias within closeness centrality could be fixed by iteratively finding and removing dependencies of the most central node, then recomputing centralities. We did this until all are zero (“unimportant”). Social network theory [17] states that two nodes connected by a mutual friend or enemy (known as a *stable triad*) will tend to become friends, and thus we defined a *dependency* of a node *i* as *i* itself plus any edges in a stable triad with *i*, illustrated by Fig. 3. In both cases if node *A* was most central we assumed edge *BC* to be *coincidental* and remove node *A* and edge *BC* before recomputing centralities. We first generalize iterative centrality using Algorithm 1, with *X* acting as a placeholder for some *backbone* algorithm.

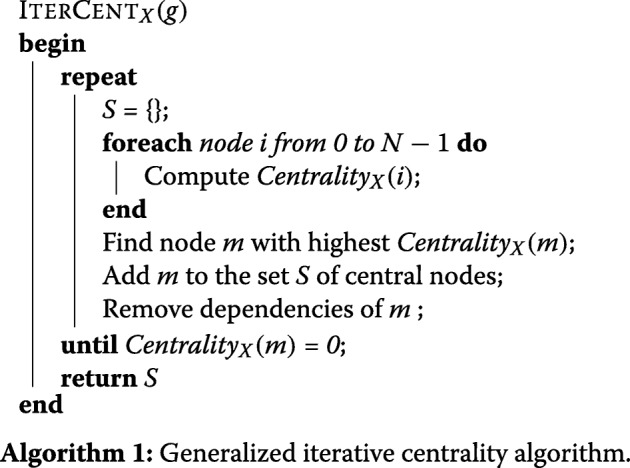


ATRIA also extended closeness centrality to operate on an undirected network with edge weights in the range [−1,1] by approaching centrality from the perspective of a node’s benefit to the network. We used a simplified economic Payment Model [18], defining closeness (*CLO*) centrality *Centrality*_*CLO*_(*i*) of node *i* by Eq. 1.

1 $$ Centrality_{CLO}(i) = | \sum_{j \ne i} G(i, j) + L(i, j)|,   $$

where *G*(*i*,*j*) is the maximum positive edge weight product over all paths between node *i* and node *j*, and *L*(*i*,*j*) is the maximum negative edge weight product. We computed these paths using a modified Dijkstra’s algorithm *MOD_DIJKSTRA* that used edge products and chose maximum path magnitudes. This is just closeness centrality using maximum paths, with “path length” defined as *G*(*i*,*j*)+*L*(*i*,*j*). Plugging *CLO* into *X* in Algorithm 1 represents our iterative closeness centrality algorithm ATRIA. We now define signed versions of other path-based backbones.

### Signed versions of other path-based approaches

#### Degree centrality

Degree is easiest to define, with all local computations. For gains and losses we count incident positive and negative edges for a node *i*, producing:

2 $$ Centrality_{DEG}(i) = |\sum_{j \ne i} W(i, j)|,  $$

where *W*(*i*,*j*) is the signed weight of edge (*i*,*j*).

#### Betweenness centrality

Betweenness is more challenging, but we can use the same *MOD_DIJKSTRA* algorithm to count the *number* of positive paths (call this *γ*_*jk*_(*i*))) and negative paths (call this *λ*_*jk*_(*i*)) that include *i*. The equation then becomes the sum of these terms:

3 $$ Centrality_{BET}(i) = \sum_{j \ne i \ne k} \gamma_{jk}(i) + \lambda_{jk}(i).  $$

We can then plug *BET* or *DEG* for *X* in Algorithm 1 to respectively produce iterative betweenness or degree centrality. Since non-iterative path-based approaches produced extremely different results on our networks, we will use these iterative versions ITERCENT _*BET*_, ITERCENT _*CLO*_, and ITERCENT _*DEG*_ to demonstrate MATRIA. Other centrality algorithms can be substituted for *X*, and we will in fact show that MATRIA can support any *k* centrality algorithms.

Table 2 shows the updated rank vector correlations for iterative path-based algorithms on our biological networks, confirming improved performance for network A before any attempt to resolve disagreements (especially for betweenness). The less modular networks B and C do not show as much improvement and are sometimes worse. We now describe MATRIA, which produces a unified ranked set that correlates well with each iterative path-based approach.

**Table 2:** Rank vector correlations between iterative path-based centrality algorithms

Algorithm Pairs	A	B	C
ITERCENT _*BET*_ XX-XX/ITERCENT _*CLO*_ XX-XX	0.498	0.208	0.060
ITERCENT _*BET*_ XX-XX/ITERCENT _*DEG*_ XX-XX	0.569	0.538	0.298
ITERCENT _*CLO*_ XX-XX/ITERCENT _*DEG*_ XX-XX	0.717	0.189	0.209

## MATria

Algorithm 2 shows our top-level MATRIA procedure that accepts a network *g* and produces the sets of central nodes *S*_*BET*_, *S*_*CLO*_ and *S*_*DEG*_, then resolves disagreements between these sets through a procedure UNIFY to produce a final set *S*.


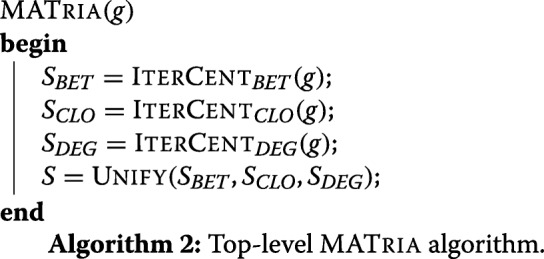


### Universal agreements

We define universal agreements as nodes discovered by all iterative backbones, or any *x*:*x*∈*S*_*BET*_∩*S*_*CLO*_∩*S*_*DEG*_. On network A the iterative backbones agreed on twelve central nodes, colored black in Fig. 1**b1-3** and labeled *A*1- *A*12. Recall this is already an improvement upon the non-iterative versions, which agreed on only five central nodes in the same vicinity. UNIFY first adds these twelve universal agreements to *S*.

### Resolving disagreements

In Fig. 1**c** we label nodes found by one or two of the path-based backbones, but not all three (18 total). We use node color to indicate the backbone(s) that discovered them, with primary colors for nodes discovered by one backbone:

- **Betweenness** (4), colored **red**: *B*1- *B*4
- **Closeness** (5), colored **yellow**: *C*1- *C*5
- **Degree** (2), colored **blue**: *D*1, *D*2

We use secondary colors obtained by combining appropriate primary colors for nodes discovered by two backbones:

- **Betweenness & Closeness** (1), colored **orange**: *BC*1
- **Closeness & Degree** (5), colored **green**: *CD*1- *CD*5
- **Betweenness & Degree** (1), colored **violet**: *BD*1

**Fig. 4:**
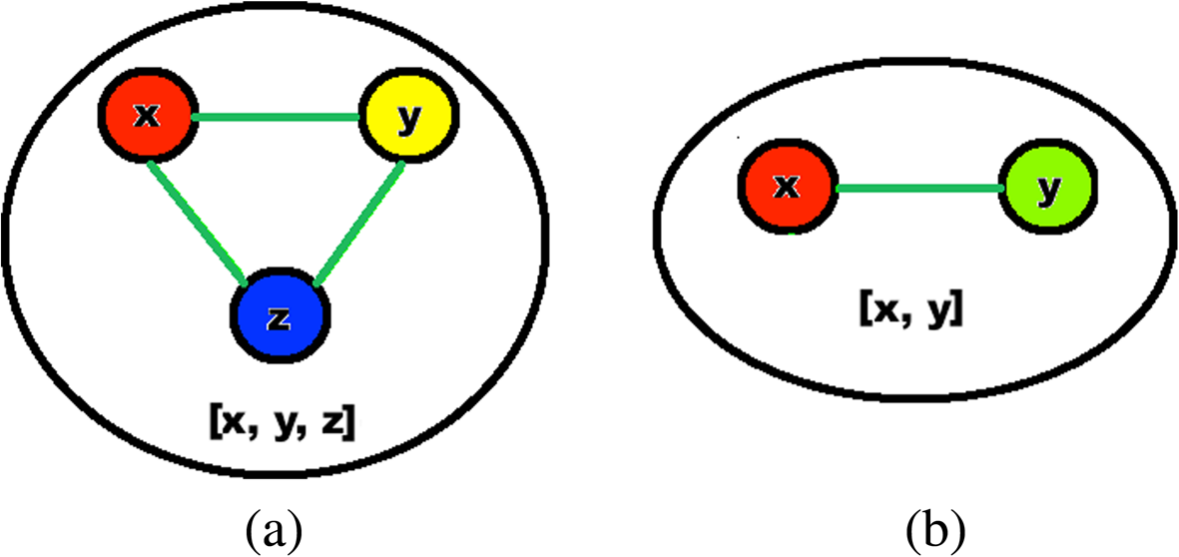
Supernode examples; (**a**) triad, (**b**) adjacency

We note patterns among these disagreements. Many times all three backbones are covered exactly once between two adjacent or three triad nodes. We argue that because of the fundamental properties of iteration, centrality is likely a “toss-up” in these situations. Take for example the triad [*x*,*y*,*z*] in Fig. 4**a**. In this case *x*, *y* and *z* were found as central by iterative betweenness, closeness and degree respectively. However, suppose centrality is actually a “toss-up” between them, which would mean for example in iterative betweenness when *x* was found as most central, *y* and *z* had only slightly lower centrality values. In the next iteration *x* would be removed along with edge *y*−*z*, causing *y* and *z* to lose all contributions from paths involving this triad (which by definition are likely significant if *x* was central). The same thing would happen when *y* was found by iterative closeness, and *z* by iterative degree. Adjacencies like the one in Fig. 4**b** have the same issue for the same reason, with *x* (or *y*) losing contributions from its central neighbor upon its removal.

We define a *supernode* as any set of neighboring nodes such that each algorithm finds exactly one of them. In Fig. 1**c** we have two supernode triads: [*B*1,*C*1,*D*1] and [*B*3,*C*5,*D*2]. UNIFY adds these to *S* (now 14 elements) as “toss-ups”, and we also darken them in our updated Fig. 1**d** to indicate they have been resolved. For supernode adjacencies there are three types: red-green (betweenness, closeness/degree), yellow-violet (closeness, betweenness/degree), and blue-orange (degree, betweenness/closeness). We have a total of six supernode adjacencies in Fig. 1**c** and begin by adding them to *S*: [*B*1,*CD*1], [*B*2,*CD*2], [*B*3,*CD*3], [*B*3,*CD*4], [*B*4,*CD*3], and [*C*2,*BD*1].

We now have an issue, because two of these adjacencies also include supernode triad members (*B*1 and *B*3). Having supernodes that share members is not helpful, because each supernode should provide multiple options for a central node. We now describe how UNIFY merges supernodes with common members, and specifically address the triad and adjacency in detail to handle this network. Supernode triads can also overlap with each other, as can supernode adjacencies, and we later briefly describe how to merge those.

#### Merging overlapping supernodes

**Fig. 5:**
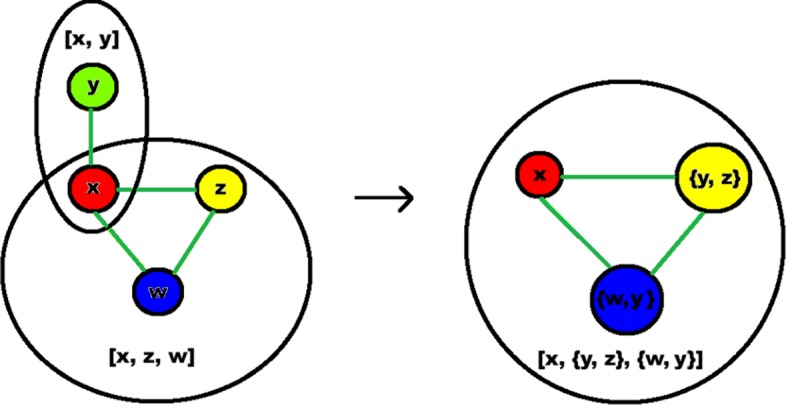
Merging supernodes; in this case an overlapping triad and adjacency

We first note that for a supernode adjacency *x*-*y*, if *x* is also a member of a supernode triad it is already a “toss up” with two nodes *w* and *z*, as shown in Fig. 5. We then note that *w* and *z* must be found by the same two algorithms that found *y* (since in a supernode triad all three algorithms must be covered). Thus, the “toss-up” becomes between (1) only *x*, (2) *y* and *w*, and (3) *y* and *z*. We merge these into one supernode triad [*x*,{*y*,*w*},{*y*,*z*}], now allowing a single node to represent a set of nodes as shown in the Figure. Although the edges from *x* to {*y*, *w*} and {*y*, *z*} now become ambiguous, their weights are no longer relevant because we already ran the backbones.

We have several supernode adjacencies in our network where one of the two nodes is also in a supernode triad:

- **Central Triad** [*B*1,*C*1,*D*1] with adjacency [*B*1,*CD*1]. We replace both elements in *S* by the supernode: [*B*1,{*C*1,*CD*1},{*D*1,*CD*1}].
- **Upper Triad** [*B*3,*C*5,*D*2] with adjacencies [*B*3,*CD*3] and [*B*3,*CD*4]. We replace all three elements in *S* by the supernode
  [*B*3,{*C*5,*CD*3,*CD*4},{*D*2,*CD*3,*CD*4}].
- **New Triad** [*B*3,{*C*5,*CD*3,*CD*4},{*D*2,*CD*3,*CD*4}] now has an overlap with adjacency [*B*4,*CD*3]. We similarly replace both elements in *S* by the supernode [{*B*3,*B*4},{*C*5,*CD*3,*CD*4},{*D*2,*CD*3,*CD*4}].

Figure 1**d** shows all resolved disagreements darkened. In addition, Table 3 shows the other types of supernode merges performed by UNIFY, between triads that share one or two nodes or adjacencies that share one. Merging provides the final set *S* in UNIFY, which we now fully write as Algorithm 3.

**Table 3:** Other types of supernode merges

Category	SN 1	SN 2	Replace Both By
Triad (2 Shared)	[*x*,*y*,*w*]	[*x*,*y*,*z*]	[*x*,*y*,{*w*,*z*}]
Triad (1 Shared)	[*x*,*v*,*w*]	[*x*,*y*,*z*]	[*x*,{*v*,*y*},{*w*,*z*}]
Adjacency(1 Shared)	[*x*,*y*]	[*x*,*z*]	[*x*,{*y*,*z*}]


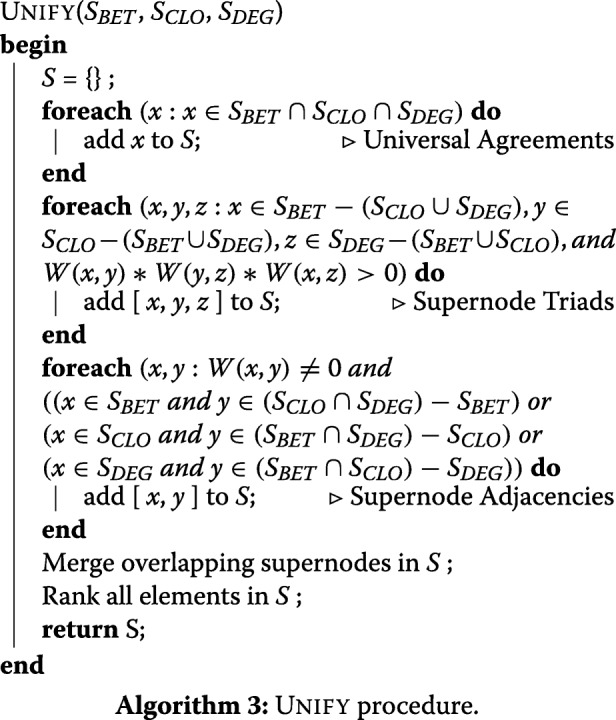


**Ranking Supernodes:** The final step of UNIFY is to rank the elements of *S*. We do this as follows:

1. **Universal Agreements:** Mean ranking over backbones.
2. **Supernode Triads:** Mean ranking of each node using the backbone that found it. For example in Fig. 4**a** we would average the ranking of *x* in betweenness, *y* in closeness, and *z* in degree.
3. **Supernode Adjacencies**: Same as supernode triads, except one node will have rankings for two backbones.
4. **Merged Supernodes**: These have elements like {*w*,*y*} where *w* and *y* were said to *both* be important by a backbone. In this case use the ranking of whichever of *w* and *y* was discovered first as the ranking of {*w*,*y*}, then apply the above logic for the supernode ranking. Our results, shown in Fig. 1**e** (red=high and violet=low rank), indicate that the top five entries (*A*1,*A*2,*A*5,*A*8, and the supernode *BD*1- *C*2) could correspond to leaders of the five most tightly connected components.

**Unresolvable Disagreements:** Although most disagreements in Fig. 1 were resolvable there are still two nodes *C*3 and *C*4 that were found by closeness and not involved in a resolvable disagreement. These are still colored yellow in Fig. 1**d**. Upon further investigation the disagreement resulted because iterative degree and betweenness found node *A*7 early (#2 and #7), but closeness found it later (#16, but more importantly after *C*3 and *C*4). With *A*7 directly connected to *C*3, removing it plummeted *C*3 in degree and betweenness centrality. But since *A*7 was also eventually discovered by closeness it became a universal agreement and could not be a supernode with *C*3. This seems to suggest forming supernodes on-the-fly, as opposed to waiting until the end. However the drop of *C*4 resulted from an indirect effect (removing *A*7 reduced many edges in that tight component), so that will not resolve all disagreements either. The other disagreement, *BC*1 and *CD*5, creates an interesting situation where two backbones each say one is important, but one (closeness) says both are important (i.e. not a “toss-up”). We leave this as unresolvable for now, though could potentially add another type of element in *S* which encapsulates this. We will see however that even with our current approach, these unresolvable disagreements are quite rare in our networks.

We also remark that UNIFY can be generalized to work with any *k* centrality algorithms. In our example (*k*=3), we can view supernode adjacencies and triads as components of size 2 and 3. In general supernodes can be of sizes 2 to *k*.

## Results

### Coverage

We begin by evaluating the percentage of nodes for which UNIFY could reach an agreement on centrality. Table 4 shows that the number of agreed important nodes did not drop significantly as our networks became less modular. While the universal agreement (important and unimportant) percentage did drop, most of these nodes became involved in supernodes, all5owing us to still draw conclusions about their centrality. Only 3-7% of nodes were involved in unresolvable disagreements, demonstrating that MATRIA will generally produce a set with good coverage.

**Table 4:** MATRIA coverage of all three networks

	A	B	C
Agreed Important	12	11	7
Agreed Unimportant	96	127	50
Supernode Triad	6	20	3
Supernode Adj	8	31	57
Unresolvable	4	14	9
% Universal Agreement	86	68	45
**% Resolvable**	**97**	**93**	**93**

We also checked some of the agreed important genes discovered by MATRIA in network B. Although gene essentiality statistics are limited for the Pacific Oyster, the results show promise. The gene for the most abundant and fundamental eukaryotic protein, Actin [19], was found and ranked #2 by MATRIA. MATRIA also found genes for Death-Associated Protein 3 (DAP3) which has been marked essential in other eukaryotic organisms for its critical roles in respiration and apoptosis [20], and the Heat Shock Protein (HSP) which has also been marked essential for apoptosis in both prokaryotes and eukaryotes [21] and is involved in protein folding [22]. Additionally, MATRIA found genes for a member of the Sterile Alpha Motif (SAM) homology, which is known to have important roles in immunity [23] and its ability to bind to RNA [24], and also a Protein-Tyrosine Phosphatase Non-Receptor (PTPN, [25]) which has potential to affect multiple cellular functions through post-translational phosphorylation [26].

### Correlations

We next verify that the rank vector for *S* correlates with the individual rank vectors *S*_*BET*_, *S*_*CLO*_, and *S*_*DEG*_, plus those found when including PN-Centrality and PageTrust (thus *k*=5). Table 5 shows that for all five examples we were able to produce a ranking with moderate and consistent correlations across all iterative backbones, with correlations tending to decrease as the network became less modular to just below 0.5 in the worst case (still demonstrating correlation).

**Table 5:** MATRIA rank vector correlations

Algorithms	A	B	C
MATRIA/ITERCENT _*BET*_ XX-XX	0.636	0.598	0.386
MATRIA/ITERCENT _*CLO*_ XX-XX	0.708	0.606	0.404
MATRIA/ITERCENT _*DEG*_ XX-XX	0.684	0.635	0.486
MATRIA/ITERCENT _*PN*_ XX-XX	0.696	0.614	0.507
MATRIA/ITERCENT _*PT*_ XX-XX	0.698	0.597	0.470

## Discussion

**Fig. 6:**
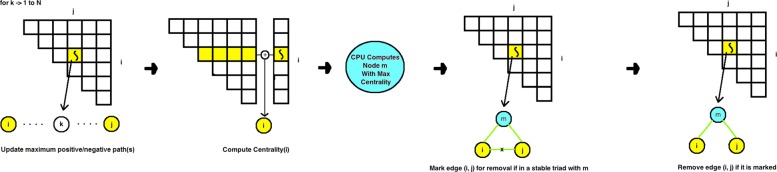
Steps for our GPU multi-threaded code, and specific operations for each thread

As we realize that iteration is computationally expensive, we parallelize MATRIA for the GPU using a four-step process demonstrated by Fig. 6. We can envision GPU threads as a jagged array indexed by two values *i* and *j*, where *i*<*j*. Each thread (*i*,*j*) first computes any maximum positive and negative paths between node *i* and node *j* in parallel. We then take *N* threads (for a network with *N* nodes), one per row, to compute the centrality of each element *i*. Next, we compute the most central node *m* on the CPU, followed by each thread (*i*,*j*) marking edge (*i*,*j*) if it (1) exists and (2) is in a stable triad with *m*. Finally each thread (*i*,*j*) removes edge (*i*,*j*) if it is marked. Table 6 shows the wall clock execution time of MATRIA on a Tesla K20 GPU, demonstrating that with this power MATRIA can practically produce results for networks in the low- to mid- thousands. Compared to serial execution on a 1.6 GHz CPU with 16 GB of RAM, this yielded 8- to 16- fold speedups on the first three networks and orders of magnitude speedups on the larger two (respectively over an hour and on pace for multiple days on the CPU). We continue to look for ways to run MATRIA on larger networks.

**Table 6:** MATRIA wall clock execution times

Sample Network	Nodes	GPU Wall Clock Time (s)
Lung Bacterial Co-Occurrence	126	0.48 +/- 0.00
Oyster Gene Co-Expression	203	1.60 +/- 0.00
Scale-Free Synthetic	500	28.52 +/- 0.03
Scale-Free Synthetic	1000	60.61 +/- 0.04
Fruit Fly Protein-Protein	2997	4357.94 +/- 3.97

## Conclusions

Our results illustrate that applying iteration to centrality algorithms with different definitions of “importance” and unifying their results gives more meaning to their computed central node sets. By resolving disagreements MATRIA produces a ranked list of central nodes and supernodes, with a cardinality much smaller than the size of the network and several mutually agreed unimportant nodes removed. Rank vectors correlate well between this set and the individual iterative backbones and are much more consistent compared to just the iterative or non-iterative backbones. While cases of unresolvable disagreements can still occur in this unified set, they are rare. Through GPU optimizations MATRIA is currently practical for medium-sized networks, and we are exploring ways to push this boundary. We also plan to experiment with weighted averages when computing overall rankings. Finally, applying MATRIA to directed (i.e. metabolic) biological networks will require an extension of iteration and supernodes to incorporate direction (i.e. adjacency *x*→*y* would now be different from *x*←*y*), an interesting question that we plan to immediately pursue.

## Abbreviations

ATria: Ablatio Triadum
GPU: Graphics Processing Unit
MATria: Multiple Ablatio Triadum
